# Gender differences in the relationship between dietary energy and macronutrients intake and body weight outcomes in Chinese adults

**DOI:** 10.1186/s12937-020-00564-6

**Published:** 2020-05-18

**Authors:** Jian Zhao, Jian Sun, Chang Su

**Affiliations:** 1 Institute of Basic Medical Sciences, Chinese Academy of Medical Sciences / School of Basic Medicine, Peking Union Medical College, Beijing, 100005 China; 2 School of Public Health & Management, Ningxia Medical University, Yinchuan, 750004 Ningxia China; 3 National Institute for Nutrition and Health, Chinese Center for Disease Control and Prevention, Number 29, Nanwei Road, Xicheng District, Beijing, 100050 China

**Keywords:** Nutritional assessment, Macronutrient, Obesity, Gender

## Abstract

**Background:**

To explore the gender differences in the relationship between dietary energy and macronutrients intake and body weight outcomes in Chinese adults.

**Methods:**

Data from the China Health and Nutrition Survey (CHNS, 2015) for10,898 participants aged 18–64 years. Three consecutive 24-h dietary recalls was used to assess the dietary intake. Quantile regression models for body mass index (BMI) and waist circumference (WC) were performed separately for each sex.

**Results:**

Adult males showed greater absolute intakes of energy and macronutrients as compared to females as per the body weight outcomes. A 10% increase in BMI resulted in an additional intake of 0.002–0.004 kcal/d of dietary energy, 0.032–0.057 g/d of fats, 0.039–0.084 g/d of proteins, and 0.018–0.028 g/d of carbohydrates across all quantiles in males (*p* <  0.05). A 10% increase in WC lead to an additional intake of 0.004–0.008 kcal/d of dietary energy, 0.051–0.052 g/d of carbohydrates across the entire quantile in males (*p* <  0.05), and an increased intake of 0.060–0.150 kcal/d of fat in females (*p* <  0.05).

**Conclusions:**

Dietary fat intake could be the risk factor of abdominal obesity in women. The importance of gender-specific evidence should be considered before promoting macronutrient allocation for the prevention and treatment of obesity.

## Introduction

Imbalanced dietary energy and macronutrient intake is associated with weight gain and increased risk of chronic non-communicable diseases that kills approximately 3 million people worldwide each year [1, 2]. According to the Chinese Residents Nutrition and Chronic Disease Status Report, the obesity rates of Chinese adults aged 18 and above increased from 4.8% in 2002 to 11.9% in 2015 [3]. Identifying effective solutions to prevent further weight gain has received attention, as it is a major public health concern in the light of the obesity epidemic in China.

Dietary energy is primarily provided by macronutrients including fats, carbohydrates, and proteins, and the cause of weight gain is energy imbalance [4]. Several preliminary studies suggested that the macronutrient composition in the diet plays an important contributory role in obesity [5, 6]. However, the role of individual macronutrients in the development of obesity remains controversial [7]. Understanding the usual intake of dietary energy and macronutrients is essential for estimating appropriate dietary intake and nutritional interventions [8]. Body mass index (BMI) and waist circumstance (WC) are the commonly used parameters to assess general and abdominal adiposity in clinical practice [9]. A few studies have been conducted on energy intake and assessment of the percentage contribution of macronutrients in Brazil, Iranian, Japan, United Kingdom, and United States [10, 11], but to some extent have lacked a comprehensive information linking dietary energy and specific macronutrients directly to general and abdominal obesity in the largest developing country, China. Additionally, sex disparities should not be overlooked in studies of overweight and obesity along with differences in physiological and lifestyle factors [12].

Using updated data from the China Health and Nutrition Survey (CHNS, 2015), the current study aimed to provide a detailed description of the sex-related distribution of dietary energy and macronutrient intake, and further investigate the possible relationship between BMI and WC with energy and the percentage of energy intake from macronutrients among Chinese adults.

## Materials and methods

### Study design and subjects

We used data of the CHNS (2015) for the present investigation, which is a large-scale, longitudinal, household-based survey initiated in 1989 that consists of representative participants of varying economic status, health indicators, and geographic areas throughout China [13]. A multistage, random cluster sample was used in the study [14]. Further information on survey procedures and the sampling scheme is reported in detail elsewhere [15]. We excluded participants who were pregnant or lactating or who reported implausible energy intakes (< 800 kcal/d or > 6000 kcal/d for males and < 600 kcal/d or > 4000 kcal/d for females). Hence, our final sample consisted of 10,898 observations (4934 males and 5964 females) aged 18–64 years with complete demographic data and information on socioeconomic status and 3-day, 24 h dietary recalls in a survey year.

### Dietary data

Well-trained interviewers asked all participants by a semi-quantitative food frequency questionnaire (FFQ) for dietary assessment. We used three consecutive 24-h dietary recalls to assess the individual levels of total energy intake [16]. We calculated the percentage of fats, carbohydrates, and proteins in the daily energy intake based on the Chinese Food Composition Table to represent the dietary structure [17]. Energy levels and macronutrient deficiencies or surpluses in the sample and age-sex subgroup were also assessed based on appropriate dietary reference intakes (DRIs) [11].

### Anthropometrics and obesity indicators

Well-trained health workers measured the height (model 206, SECA), weight (model 880, SECA), and WC of participants following standardized procedures. We calculated BMI by dividing the weight (in kg) by the square of the height (in m^2^). We grouped the BMI into underweight (< 18.5 kg/m^2^), normal (18.5–23.9 kg/m^2^), overweight (24–27.9 kg/m^2^), and obesity (> 28 kg/m^2^), based on the recommended cut-off points of BMI for overweight and obesity in Chinese adults by the Working Group on Obesity in China [18]. We measured WC from the midpoint between the lower border of the rib cage and the iliac crest to the nearest 0.1 cm using a SECA tape. We defined participants as having abdominal obesity if the WC ≥85 cm in females and ≥ 90 cm in males in accordance with the guideline of the National Health and Family Commission for Chinese Adults (2013, [19]).

### Other relevant variables

We grouped participants into two age groups (18–44 and 45–64 years), two marital statuses (single and married), three education level (primary/illiterate, middle, and high school/above), two geographical regions (rural and urban), and three income levels (low, medium, and high). We classified the smoking status and drinking status as current or ever/never. Physical activity (PA) included four domains: occupational, household chore, leisure time, and transportation activities. Participants reported all PAs in average hours per week, and we converted the time spent in each activity into a metabolic equivalent of task (MET) hours per week based on the Compendium of Physical Activities. We grouped the total MET hours per week into low, middle, and high [20].

### Statistical analysis

Statistical analyses were performed with SAS 9.4 (SAS Institute, Inc. Cary, NC, USA). The values were reported as mean and standard errors for continuous variables or as proportions of the total for categorical variables. Descriptive statistics for sample characteristics were presented as weighted mean or weighted percentage. The F-test (continuous variables) and adjusted Pearson chi-square tests (categorical variables) were used to determine sex-based differences in the distribution of dietary intake with basic characteristic and body weight outcomes. Using the PROC QUANTREG procedure, series quantile regression models for continuous BMI and WC measurements in males and females were conducted to explore the associations between energy intake and macronutrients composition for general and abdominal obesity in China. Two models were tested that included an unadjusted model (model 1) and an adjusted model (model 2), where the latter controlled for additional individual variables including age, education level, income level, geographic region, physical activity, drinking, and smoking.

## Results

### Basic characteristics of the study population

Descriptive differences of basic characteristics of the participants were presented in Table 1. Of the 10,898 adult participants, 4934(45.3%) were males and 5964(54.7%) were females. The average BMI and WC was 24.4 kg/m^2^ and 82.6 cm in males, 24.0 kg/m^2^ and 81.0 cm in females, respectively. The intake of dietary energy, fats, proteins, and carbohydrates was 2273.9 kcal/d, 91.5 g/d, 74.0 g/d, and 282.4 g/d in males, and 1919.4 kcal/d, 77.7 g/d, 62.3 g/d, and 242.0 g/d in females, respectively. Some variables were observed to have statistically significant differences between the sexes, such as age, income, smoking, drinking, energy and macronutrients intake (*p* <  0.001). Males consumed more dietary energy, fats, proteins, carbohydrates than females (*p* <  0.001). In addition, males had higher BMI and WC than females (*p* <  0.001).

**Table 1:** Baseline characteristics of the study population in CHNS(2015) by genders^a,b^

General Characteristic	Women(*N* = 5964)	Men(*N* = 4934)	*p*-Value
Age,*n* (*%*)			< 0.001
18–44 years	2318(38.9)	1753(35.5)	
45–64 years	3646(61.1)	3181(64.5)	
Education level, *n* (*%*)			< 0.001
Primary/illiterate	1752(29.4)	933(18.9)	
Middle school	2122(35.6)	1929(39.1)	
High/above	2090(35.0)	2072(42.0)	
Income level, *n* (*%*)			0.005
Low	1883(31.6)	1429(29.0)	
Medium	1963(32.9)	1630(33.0)	
High	2118(35.5)	1875(38.0)	
Geogerphical region, *n* (*%*)			0.437
Rural	3727(62.5)	3119(63.2)	
Urban	2237(37.5)	1815(36.8)	
PA level, *n* (*%*)			< 0.001
Low	1923(32.2)	1708(34.6)	
Medium	2108(35.3)	1536(31.1)	
High	1933(32.4)	1690(34.3)	
Smoking, *n* (*%*)			< 0.001
Ever/Never	5870(98.4)	2155(43.7%)	
Current	94(1.6)	2779(56.3%)	
Drinking, *n* (*%*)			< 0.001
Ever/Never	5563(93.3)	2153(43.6)	
Current	401(6.7)	2781(56.4)	
BMI category, *n* (*%*)			< 0.001
Underweight	291(4.9)	183(3.7)	
Normal	2931(49.1)	2220(45.0)	
Overweight	1933(32.4)	1820(36.9)	
Obesity	809(13.6)	711(14.4)	
BMI (kg/m^2^)	24.0 ± 0.1	24.4 ± 0.1	< 0.001
WC (cm)	81.0 ± 0.2	86.2 ± 0.2	< 0.001
Dietary intake			
Daily energy (k cal)	1919.4 ± 8.9	2273.9 ± 11.3	< 0.001
Fat (g)	77.7 ± 0.6	91.5 ± 0.7	< 0.001
Protein (g)	62.3 ± 0.3	74.0 ± 0.4	< 0.001
Carbohydrate (g)	242.0 ± 1.4	282.4 ± 1.8	< 0.001
Fat (% E^3^)	35.9 ± 0.2	36.0 ± 0.2	0.819
Protein (% E ^4^)	13.2 ± 0.1	13.2 ± 0.1	0.197
Carbohydrate (% E^5^)	50.7 ± 0.2	49.8 ± 0.2	< 0.001

^a^Data for categorical variable expressed as number (%); ^b^Values are mean ± s.e. for continuous variables. % E^3,4,5^ means the percentage of energy intake from fat, protein and carbohydrate, respectively

### Dietary intake by social demographic characteristics

The distribution of dietary intake by social demographic characteristics in different genders were presented in Table 2. There were significant differences in the dietary energy intake between different income levels and PA groups in females (*p* <  0.001), and between different geogerphical regions and PA groups in males (*p* <  0.001). Participants with the highest income had the most dietary fat intake regardless of gender (94.9 g in males and 80.5 g in females; *p* <  0.001). Females aged 18–44 years (62.5 g) had more dietary protein intake than females aged 45–64 years (61.8 g) (*p* <  0.001). Additionally, there were significant differences in dietary protein intake among different education levels, income levels, and geogerphical regions regardless of gender (*p* <  0.001). Urban males had the highest protein intake of 77.4 g. Dietary carbohydrate intake varied significantly across social demographic characteristics. The highest levels of PA had the most carbohydrate intake regardless of gender (296.3 g in males and 256.6 g in females; *p* <  0.001).

**Table 2:** The distribution of daily energy and macronutrient intake by social-demographic characteristics in 2015^a,b^

General Characteristic	Women (*N* = 5964)				Men (*N* = 4934)			
	Energy (k cal)	Fat(g)	Protein(g)	Carbohydrate(g)	Energy (k cal)	Fat(g)	Protein(g)	Carbohydrate (g)
Age								
18–44 years	1931.4 ± 14.5	77.8 ± 0.9	62.5 ± 0.5 ^**^	244.5 ± 2.2^*^	2300.6 ± 19.5	91.8 ± 1.2	74.7 ± 0.7	290.8 ± 3.2 ^***^
45–64 years	1911.7 ± 11.3	77.7 ± 0.7	61.8 ± 0.4	240.4 ± 1.7	2259.2 ± 13.9	91.3 ± 0.9	73.6 ± 0.5	277.7 ± 2.1
Education level								
Primary/illiterate	1915.3 ± 16.4	76.5 ± 1.0	59.0 ± 0.6^***^	246.9 ± 2.6^***^	2258.7 ± 26.2	90.8 ± 1.6	69.2 ± 0.9^***^	283.5 ± 4.3 ^***^
Middle school	1938.8 ± 15.6	77.3 ± 1.0	61.5 ± 0.6	248.4 ± 2.5	2297.1 ± 19.1	91.1 ± 1.1	73.6 ± 0.7	288.5 ± 3.0
High/above	1903.0 ± 14.5	79.2 ± 0.6	65.4 ± 0.6	231.3 ± 2.0	2259.1 ± 16.5	92.1 ± 1.4	76.6 ± 0.6	276.1 ± 2.5
Income level								
Low	1964.4 ± 17.4^***^	78.8 ± 1.1^***^	60.8 ± 0.6^***^	251.9 ± 2.7^***^	2300.9 ± 22.4	90.9 ± 1.3^***^	71.4 ± 0.7^***^	293.4 ± 3.7 ^***^
Medium	1894.7 ± 15.0	73.7 ± 0.9	60.4 ± 0.5	246.6 ± 2.5	2260.3 ± 19.7	88.1 ± 1.2	72.4 ± 0.7	287.7 ± 3.2
High	1903.1 ± 14.1	80.5 ± 1.0	64.8 ± 0.5	228.9 ± 2.0	2265.1 ± 17.5	94.9 ± 1.1	77.4 ± 0.7	269.3 ± 2.5
Geogerphical region								
Rural	1914.9 ± 11.3	74.9 ± 0.7^***^	59.9 ± 0.4^***^	249.4 ± 1.8 ^***^	2295.2 ± 14.5^**^	90.4 ± 0.9^***^	72.4 ± 0.5^***^	290.9 ± 2.3 ^***^
Urban	1927.0 ± 14.6	82.4 ± 1.0	65.8 ± 0.6	229.6 ± 2.0	2237.2 ± 18.0	93.3 ± 1.1	76.8 ± 0.7	267.6 ± 2.7
PA level								
Low	1892.7 ± 16.0^***^	77.8 ± 1.1	61.9 ± 0.6	235.4 ± 2.4^***^	2258.8 ± 19.8^***^	91.7 ± 1.2	73.7 ± 0.7	279.0 ± 3.0^***^
Medium	1890.9 ± 14.4	77.7 ± 0.9	62.3 ± 0.5	234.6 ± 2.2	2221.8 ± 18.8	90.8 ± 1.1	74.3 ± 0.7	270.7 ± 3.0
High	1976.9 ± 16.0	77.5 ± 1.0	62.1 ± 0.6	256.6 ± 2.6	2336.4 ± 20.0	91.9 ± 1.2	74.0 ± 0.7	296.3 ± 3.2

^a^Values are mean ± s.e. for continuous variables .^b^ ***, ** and * indicate statistical significance at the 1, 5 and 10% level, respectively

There were significant differences in the distribution of dietary energy from macronutrients as per BMI categories by sex differentiation (Table 3). Males showed greater absolute intakes of dietary energy and macronutrients as compared to females in the BMI categories comparison (*p* <  0.001). In the normal BMI category, the percentage of dieary energy intake from carbohydrate in females (50.7%) was significantly higher than that in males (49.9%) (*p* <  0.001). In the overweight BMI category, the percentage of dieatry energy intake from carbohydrates in females (50.6%) was significantly higher than that in males (49.5%), while the percentage of dietary energy intake from proteins in females (13.1%) was significantly lower than that in males (13.4%)(*p* <  0.001).

**Table 3:** Dietary energy and macronutrient intake among different BMI categories by gender in 2015^a,b^

Dietary intake	The underweight BMI category		The normal BMI category		The overweight BMI category		The obesity BMI category	
	Women (*N* = 291)	Men (*N* = 183)	Women (*N* = 2931)	Men (*N* = 2220)	Women (*N* = 1933)	Men (*N* = 1820)	Women (*N* = 809)	Men (*N* = 711)
Energy (k cal)	1887.7 ± 40.0	2296.8 ± 65.7^***^	1908.0 ± 12.7	2234.7 ± 16.8^***^	1948.2 ± 16.1	2290.3 ± 18.4^***^	1903.2 ± 23.1	2348.2 ± 30.2^***^
Fat (g)	76.8 ± 2.7	92.4 ± 4.1^***^	77.0 ± 0.8	90.0 ± 1.1^***^	79.0 ± 1.0	92.4 ± 1.1^***^	77.4 ± 1.6	93.6 ± 1.8^***^
Protein (g)	60.6 ± 1.4	71.4 ± 2.1^***^	62.1 ± 0.5	72.2 ± 0.6^***^	62.9 ± 0.6	75.5 ± 0.7^***^	60.7 ± 0.8	76.6 ± 1.1^***^
Carbohydrate (g)	237.9 ± 6.0	290.0 ± 10.3^***^	240.5 ± 1.9	277.7 ± 2.6^***^	245.0 ± 2.5	282.6 ± 2.9^***^	240.1 ± 3.4	294.1 ± 4.7^***^
Fat (% E^3^)	35.9 ± 0.7	36.1 ± 1.0	35.8 ± 0.2	35.9 ± 0.3	36.1 ± 0.3	36.1 ± 0.3	35.9 ± 0.4	35.6 ± 0.4
Protein (% E^4^)	13.0 ± 0.2	12.8 ± 0.2	13.3 ± 0.1	13.2 ± 0.1	13.1 ± 0.1	13.4 ± 0.1^**^	13.2 ± 0.1	13.0 ± 0.1
Carbohydrate (% E^5^)	50.9 ± 0.7	50.4 ± 1.0	50.7 ± 0.2	49.9 ± 0.3 ^**^	50.6 ± 0.3	49.5 ± 0.3^**^	51.0 ± 0.4	50.3 ± 0.5

^a^Values are mean (s.e.) for continuous variables and n (%) for categorical variable.^b^ ***, ** and * indicate statistical significance at the 1, 5 and 10% level, respectively. % E^3,4,5^ means the percentage of energy intake from fat, protein and carbohydrate, respectively

**Fig. 1:**
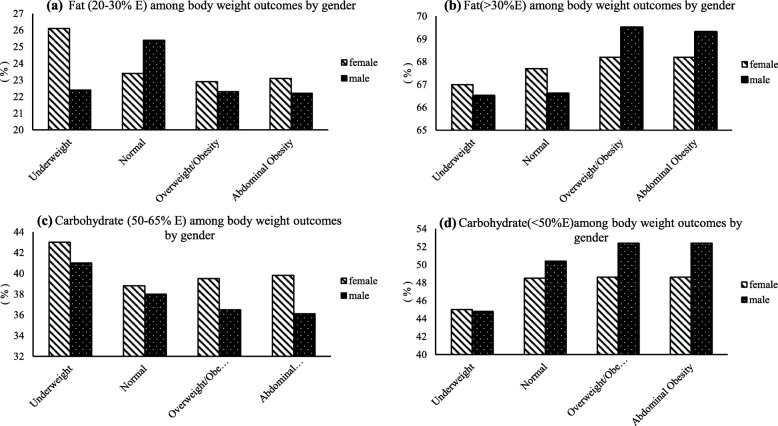
Dietary energy intake from fat and carbohydrate compared with the DRIs standard in subgroups with different weight outcomes. **a** The proportion of dietary energy intake from fat within the recommended values (20–30%) among body weight outcomes by sex. **b** the proportion of dietary energy intake from fat beyond the recommended values (> 30%) among body weight outcomes by sex. **c** the proportion of dietary energy intake from carbohydrate within the recommended values (50–65%) among body weight outcomes by sex. **d** the proportion of dietary energy intake from carbohydrate below the recommended values (< 50%) among body weight outcomes by sex

Figure 1a shows a significant difference in the percentage of dietary energy intake from fat between males and females within the DRIs recommended range (20–30%). Females in the underweight, overweight, obesity BMI categories, and abdominal obesity groups had significantly higher percentage of dietary energy intake from fat than males, in line with the DRIs (20–30%) (*p* <  0.001). Moreover, with weight gain in females, the percentage of dietary energy intake from fat was significantly lower than the DRIs recommended range (20–30%) (*p* <  0.001). The proportion of percentage of dietary energy intake from fat over the DRIs standard (> 30%) was significantly different between males and females (Fig. 1b). Specifically, in the underweight and the normal BMI category, females who exceeded the DRI standard (> 30%) was significantly more than that of males, as opposed to the overweight/obese BMI categories and abdominal obesity groups (*p* <  0.001). There was a significant difference in males and females with respect to the percentage of dietary energy intake from carbohydrates in line with the DRIs standard (50–65%) (Fig. 1c). Females in the normal, overweight and obesity BMI categories, and the abdominal obesity groups had significantly higher percentage of dietary energy intake from carbohydrates than males, in line with the DRIs (50–65%), as opposed to the underweight group. Figure 1c suggested that the proportion of the percentage of dietary energy intake from carbohydrates below the recommended DRIs (< 50%) increased significantly with weight gain (*p* <  0.001). Furthermore, males below the recommended DIRs (< 50%) (Fig. 1d) were significantly more than that of females in the normal, overweight and obesity BMI categories, and abdominal obesity groups, as opposed to the underweight group (*p* <  0.001).

As shown in Table 4, the associations between dietary energy and macronutrient consumption and BMI were estimated using quantile regressions. From the adjusted model for males, significant coefficients for BMI were observed at the 25 th, 50 th, 75 th, and 95 th dietary energy quantiles (*p* <  0.05), at the 75th and 95 th dietary fat quantiles (*p* <  0.05), at the 5 th, 25 th, 50 th, and 75 th dietary protein quantiles (*p* <  0.05), and at the 75th and 95th dietary carbohydrate quantiles (*p* <  0.05). Furthermore, the increase in dietary energy and fat intake was higher at the upper end of the distribution, suggesting that males with higher BMI had an additional dietary energy and fat intake than individuals with lower BMI. Moreover, a 10% increase in BMI lead to an additional intake of 0.002–0.004 kcal/d of dietary energy, 0.032–0.057 g/d of dietary fat, 0.039–0·084 g/d of dietary protein, and 0.018–0.028 g/d of dietary carbohydrate across all quantiles in males (*p* <  0.05).

**Table 4:** Quantile regression estimates for the association between energy intake and macronutrient composition and BMI (kg/m^2^) in CHNS 2015^a,b,c^

Dietary intake		Men					Women				
		5th	25th	50th	75th	95th	5th	25th	50th	75th	95th
Energy (kcal)	Model 1	0.0001	0.0001^*^	0.0025^**^	0.0026^***^	0.0046^***^	0.0001	0.0001	0.0001	− 0.0001	0.0001
	Model 2	0.0001	0.0002^**^	0.0003^**^	0.0004^***^	0.0004^**^	0.0001	0.0001	0.0001	0.0001	−0.0001
Fat (g)	Model 1	0.0023	0.0016	0.0031	0.0025	0.0049^***^	0.0009	0.0014	0.0014	−0.0017	−0.0007
	Model 2	0.0030	0.0020	0.0022	0.0032^***^	0.0057^***^	0.0021	0.0010	0.0010	0.0006	−0.0035
Protein (g)	Model 1	0.0056^*^	0.0100^***^	0.0107^***^	0.0085^***^	0.0088	0.0033	0.0022	0.0016	−0.0044	−0.0056
	Model 2	0.0039^**^	0.0059^**^	0.0086^***^	0.0084^***^	0.0081	0.0031	0.0027	0.0025	−0.0001	− 0.0001
Carbohydrate (g)	Model 1	−0.0003	0.0004	0.0009^***^	0.0013^***^	0.0025^**^	0.0002	0.0005	0.0007	−0.0005	− 0.0003
	Model 2	0.0001	0.0009	0.0016^*^	0.0018^**^	0.0028^***^	0.0003	0.0007	0.0001	0.0004	−0.0001
Fat (% E^4^)	Model 1	0.0009	0.0005	0.0013	0.0020	−0.0099	0.0001	0.0001	0.0030	−0.0020	−0.0021
	Model 2	0.0015	0.0006	0.0039	0.0022	0.0133	0.0071	−0.0003	0.0044	−0.0015	− 0.0150
Protein (% E^5^)	Model 1	0.0489^**^	0.0565^***^	0.0427^**^	0.0202^*^	−0.0593^**^	0.0155	−0.0045	− 0.0242	− 0.0410	−0.0520
	Model 2	0.0256	0.0180	0.0069	0.0172	−0.0623	0.020	0.0156	0.0060	−0.0160	− 0.0373
Carbohydrate(% E^6^)	Model 1	−0.0152	−0.0043	− 0.0024	0.0014	0.0028	−0.0040	− 0.0010	− 0.0005	0.0043	0.0042
	Model 2	−0.0019	0.0007	0.0040	0.0024	0.0173	−0.0066	0.0003	−0.0050	0.0036	0.0164

^a^ Model 1 included energy intake and macronutrient composition in the quantile regression model to investigate the association with BMI. ^b^ Model 2 adjusted for age (18-44y and 45-64y), education (primary/illiterate, junior and high school/above), income (low, medium and high), region (urban/rural) and physical activity (low, medium and high), current smoker (yes/no), current drinker (yes/no).^c^ ***, ** and * indicate statistical significance at the 1, 5 and 10% level, respectively. % E^4, 5, 6^ means the percentage of energy intake from fat, protein and carbohydrate, respectively

With respect to our findings stated in Table 5, significant coefficients for WC in males were observed at the 75th and 95th dietary energy quantiles (*p* <  0.05) and at the 75 th and 95 th dietary protein quantiles (*p* < 0.05), whereas, in females, the statistically coefficients were observed at the 25 th and 50 th quantiles dietary carbohydrate quantiles (*p* < 0.05). A 10% increase in WC lead to an additional intake of 0.004–0.008 kcal/d of dietary energy and 0.051–0.052 g/d of dietary carbohydrates across all quantiles in males (*p* < 0.05), and 0.060–0.150 kcal/d of dietary fat in females (*p* < 0.05). On the other hand, a 10% increase in WC lead to a reduced intake of 0.051–0.052 g/d of dietary carbohydrate across all quantiles in females (*p* < 0.05).

**Table 5:** Quantile regression estimates for the association between energy intake and macronutrient composition and WC (cm) in CHNS 2015^a,b,c^

Dietary intake		Men					Women				
		5th	25th	50th	75th	95th	5th	25th	50th	75th	95th
Energy (kcal)	Model 1	−0.0008	− 0.0001	0.0005	0.0007^**^	0.0011^**^	−0.0012	−0.0001	− 0.0001	−0.0005	− 0.0004
	Model 2	−0.0006	0.0004^*^	0.0004^*^	0.0007^**^	0.0008^**^	−0.0014	−0.0001	− 0.0002	−0.0003	− 0.0001
Fat (g)	Model 1	−0.0103	0.0056^*^	0.0086^**^	0.0041	0.0062	−0.0190	0.0010	0.0001	0.0072	0.0140
	Model 2	−0.0027	0.0082	0.0041	0.0050	0.0045	−0.0117	0.0001	0.0005	0.0060^*^	0.0150^**^
Protein (g)	Model 1	−0.0200	0.0139^**^	0.0194^***^	0.0170^**^	0.0238	−0.0500	−0.0166	− 0.0061	−0.0189	− 0.0217
	Model 2	−0.0238	0.0107	0.0105	0.0131	0.0252	−0.0385	0.0001	−0.0084	−0.0108	− 0.0098
Carbohydrate (g)	Model 1	−0.0044	−0.0001	− 0.0001	0.0042^*^	0.0064^*^	−0.0061	− 0.0007	−0.0001	− 0.0006	0.0034
	Model 2	−0.0049	0.0009	0.0023	0.0052^**^	0.0051^**^	−0.0085	−0.0001^**^	− 0.0023^**^	0.0001	0.0045
Fat (% E^4^)	Model 1	0.0226	0.0001	0.0001	0.0236	−0.0308	0.0265	0.0050	0.0001	−0.0300	−0.0515
	Model 2	0.0080	0.0069	0.0027	0.0374	−0.0323	0.0257	0.0001	0.0126	−0.0269	−0.0896
Protein (% E^5^)	Model 1	0.1543	0.0714	0.0960	0.0001	0.0810	−0.1222	−0.0974	− 0.066	−0.103	− 0.136
	Model 2	0.0367	0.0307	−0.0335	−0.0778	0.0235	0.0001	0.0001	−0.0246	0.0001	−0.075
Carbohydrate(% E^6^)	Model 1	−0.0195	−0.0001	− 0.0161	0.0211	0.0001	−0.0226	− 0.0021	−0.0001	0.0414	0.0614
	Model 2	−0.0172	−0.0020	0.0111	0.0364	0.0267	−0.0302	−0.0001	− 0.0115	0.0302	0.0897

^a^Model 1 included energy intake and macronutrient composition in the quantile regression model to investigate the association with WC. ^b^Model 2 adjusted for age (18-44y and 45-64y), education (primary/illiterate, junior and high school/above), income (low, medium and high), region (urban/rural) and physical activity (low, medium and high), current smoker (yes/no), current drinker (yes/no).^c^ ***, ** and * indicate statistical significance at the 1, 5 and 10% level, respectively. % E^4, 5, 6^ means the percentage of energy intake from fat, protein and carbohydrate, respectively

## Discussion

It is an effort to assess the latest nutritional status concentrating on the socioeconomic status and body weight outcomes. Additionally, the present study provided more evidences on sex disparities with respect to the associations between energy and macronutrient intakes and body weight outcomes in Chinese adults. Both developed and developing countries had reported sex-related differences in obesity [21, 22]. Research by the Institute for Health Metrics and Evaluation demonstrated that adult females are consistently more obese than males in developing countries, possibly due to different roles, social status, and gender-norms in the family. Additionally, a study of sex-related differences of obesity-related alterations in intrinsic brain activity showed a stronger relationship between increased BMI and decreased connectivity of core reward network components with cortical and emotional regulation regions in females, which could be related to the greater prevalence of emotional eating [23]. Therefore, it is important to consider individual sex differences to prevent and treat obesity. Our study report sex-related disparities in energy and macronutrients intake. Significant relationships were found between dietary energy intake and different income levels just in females, which illustrated that socioeconomic factors possibly induced different effects on males and females. Income was reported as an important factor to affect the dietary structure of adults in China in a previous study, which possibility influenced the purchasing power and determined the choice of food of the residents [24]. This creates cost and accessibility barriers for healthy food choices for low-income individuals [25]. Our study specifically found that females with low income had the highest dietary energy intake. Therefore, it is important to guide them to conduct reasonable energy consumption. Both western countries and China had investigated the interactions between PA and obesity risk across sexes. Rashad found that leisure time PA had a negative impact on BMI, and the effect was more pronounced in females, using nationally representative longitudinal data from Canada’s National Population Health Survey [26]. Hongqiu Gu evaluated the PA patterns of urban and rural dwellers in China and found that urban males were less physically active than rural males and had a higher prevalence of obesity in the study involving Chinese [27]. Our study reported that rural adult with high PA consumed more dietary energy, possibly due to higher energy expenditure from outdoor activities and agricultural work [28]. Additionally, from the adjusted quantile regression model, it was reported that more dietary energy intake had positive effects on the BMI and WC in males, indicating that obese male may need more energy to maintain weight. Results from the World Health Organization Multinational Monitoring of Trends and Determinants in Cardiovascular Disease aggregate level analyses further supported this positive correlation between dietary energy intake and weight status in European countries [29].

The role of dietary fat as a major determinant of obesity was not without controversy, but was better established [30]. Notably, urban residents with higher income tended to consume more dietary fat, and this was supported by previous studies reporting a positive link between fat intake, economic growth, and nutrition transitions globally in the past several decades [31]. Our study reported that more dietary fat intake had positive effects on the higher distribution of BMI (in males) and WC (in females), which implied that dietary fat could increase the risk of general obesity in males and abdominal obesity in females. A cohort study from the National Health and Nutrition Examination Survey found that the percentage of dietary energy intake from fat and weight changes were inversely related in females, but positively associated in males without any morbidity [32]. In the present study, we found that that high-fat diets are associated with greater relative body weight in adults, which was consistent with a previous meta-analysis [33]. The effect of dietary carbohydrates in weight loss had received considerable attention in light of the current obesity epidemic [34]. Researches over the last decade reported that low-carbohydrate diets were a viable option in the treatment of obesity, but its long-term effect (12 months) remained controversial [35]. Our results reported that in most socio-demographic indicators, particularly those of low socioeconomic status groups, males consumed significantly more carbohydrates than females. Previous studies had reported that low-income groups consumed more cereals and lesser fish, meat, vegetables, and fruits than high-income groups, leading to high intake of carbohydrate and lower intake of protein, fat, potassium, and vitamins. Furthermore, food shoppers with low levels of education and income were least likely to purchase foods that were comparatively higher in fiber and lower in fat [36]. Our studies also indicated that the association between carbohydrate intake and body weight outcomes showed higher magnitude in the upper tail of the BMI and WC distribution in Chinese males. Previous research reported that carbohydrates could promote the development of small intestinal bacterial overgrowth in obesity. Meanwhile, certain carbohydrate types could be related to body weight outcomes, because carbohydrates have been traditionally classified as simple or complex on the basis of their chemical structure. Further researches are needed to deepen the understanding of the relationship between dietary carbohydrate types and body weight outcomes. In the present study, urban residents with high socioeconomic status consumed more proteins. Meanwhile, the association between dietary protein and body weight outcomes showed higher magnitude in the BMI distribution, consistent with a systematic review and meta-analysis that was conducted to assess the benefits and drawbacks of high-protein diets as compared to low-protein diets, and showed that higher-protein diets probably improved adiposity [37]. The Chinese dietary guideline (CDG) version of 2016 for Chinese adults suggested the dietary energy intake from fat, carbohydrate, and protein were below 30%, 55–65%, and 10–15%, respectively. Compared with the CDG recommendations, the proportion of dietary energy intake from protein was found to be in line with the CDG recommendations, while carbohydrates were below the recommendations and fats exceeded the recommendations. Moreover, the percentage of overweight/obese people deriving the percentage of dietary energy intake from fat was more than 30%, which was significantly higher than those of normal-weight people. Moreover, the association between the percentage of dietary energy intake from fat, protein, carbohydrate and body weight outcomes were not found, which was similar to the study results conducted in Australian children and adolescents [38].

### Strengths and limitations

The present study had certain strengths. First, we used updated data to observe the distribution of dietary energy and macronutrient intake in the largest developing country, which provided the latest nutritional status of Chinese residents. Second, the sample size was large with a wide age range, and the staff was trained in the study’s methodology and standardization in different parameters at the same time by the same scientists. However, there were several limitations. First, this study was subject to the same limitations that affect all cross-sectional analysis, including possible reverse causality, therefore, extrapolated conclusion should be made cautiously. Second, observations in the survey may include samples with weight gain, weight plateau, and weight loss, which was not focus on the gender difference in plateau period of losing weight for overweight/obesity people. Third, this study lacked laboratory monitoring data, such as obesity-related alterations in intrinsic brain activity and connectivity, which were used to progress the inconsistent consideration of sex differences in underlying mechanisms. Forth, dietary data were collected using three consecutive 24-h dietary recalls, which might show relatively limited variations for a participant as compared to non-consecutive 24-h recalls. However, the average intake over 3 days could offer a relatively valid estimate of nutrient intake, as shown in an earlier study using the CHNS data.

## Conclusions

Chinese adults tend to have a high-fat and low-carbohydrate diet. Excessive intake of dietary fat could be risk factors of abdominal obesity among Chinese females. Health professionals should consider the weight of evidence before promoting one form of macronutrient distribution over another to prevent and treat obesity in different gender groups.

## Data Availability

The datasets used and analysed during the current study are available from the corresponding author on reasonable request.

## Abbreviations

CHNS: China Health and Nutrition Survey
BMI: Body mass index
WC: Waist circumference
QR: Quantile regression
CDC: Centers for Disease Control and Prevention
MET: Metabolic equivalents of task
WHO: World Health Organization
CDG: Chinese dietary guideline
SD: Standard deviation
DRI: Dietary Reference Intake
MET: Metabolic Equivalent of Task
PA: Physical activity
